# Supplementary material for: Evaluating the impact of e-registration and mHealth on institutional delivery in hazard-prone areas of Bangladesh: A protocol for a non-randomized controlled cluster trial
Source: PLoS One. 2023 Sep 27;18(9):e0271364. doi: 10.1371/journal.pone.0271364 (PMC10529544; doi:10.1371/journal.pone.0271364)
Supplement: S1 File — (PDF) [file pone.0271364.s001.pdf]

NORTH SOUTH UNIVERSITY
Institutional Review Board/ Ethics Review Committee (IRB/ERC)

ADM 625, Plot: 15, Block: B,
Bashundhara, Dhaka-1229,
Bangladesh.
PABX: +88-02-55668200, Ext: 6465

Memorandum

2022/OR-NSU/IRB/0205

Ref: 2022/OR-NSU/IRB/0205

Date: 22 March 2022

To: Dr. Ahmed Hossain
Professor
Department of Public Health

From: Professor Dipak Kumar Mitra
Chairman
NSU Institutional Review Board/ Ethics Review Committee

Chairman
NSU Institutional Review Board/
Ethics Review Committee (IRB/ERC)

Subject: Approval of Research Protocol #2022/OR-NSU/IRB/0205

Dear Dr. Ahmed Hossain,

Thank you for your application requesting for approval of your research protocol #2022/OR-NSU/IRB/0205, titled "The effect of e-Registration and mHealth on institutional deliveries in the hazard-prone areas of Southern Bangladesh: A protocol for an open-label two-arm non-randomized controlled cluster trial?". I am glad to inform you that the committee has approved your research protocol. You will be required to observe the following terms and conditions in implementing the research protocol:

1. As principal investigator, the ultimate responsibility for scientific and ethical conduct including the protection of the rights and welfare of study participants vest upon you. You shall also be responsible for ensuring competence, integrity, and ethical conduct of other investigators and staff directly involved in the research protocol.

2. You shall conduct the activity in accordance with the IRB-approved protocol and shall fully comply with any subsequent determinations by IRB.

3. You shall obtain prior approval from the IRB for any modification in the approved research protocol and/or approved consent form(s), except in case of emergency to safeguard/eliminate apparent immediate hazards to study participants. Such changes must immediately be reported to the IRB Chairman.

4. You shall recruit/enroll participants for the study strictly adhering to the criteria mentioned in the approved research protocol.

5. You shall obtain legally effective informed consent (i.e. consent should be free from coercion or undue influence) from the selected study participants or their legally responsible representative, as approved in the protocol, using the approved consent forms prior to their enrollment in the study. Before obtaining consent, all prospective study participants must be adequately informed about the purpose(s) of the study, its methods and procedures, and also what would be done if they agree and also if they do not agree to participate in the study. They must be informed that their participation

ADM 625, Plot: 15, Block: B,
Bashundhara, Dhaka-1229,
Bangladesh.
PABX: +88-02-55668200, Ext: 6465

in the study is voluntary and that they can withdraw their participation any time without prejudice. Used consent form should be preserved for a period of at least three years following official termination of the study.

6. You shall promptly report the occurrence of any Adverse Event or Serious Adverse Event or unanticipated problems of potential risk to the study participants or others to the ERC in writing within 24 hours of such occurrences.
7. Any significant new findings, developing during the course of this study that might affect the risks and benefits and thus influence either participation in the study or continuation of participation should be reported in writing to the participants and the IRB.
8. Data and/or samples should be collected, as specified in the IRB-approved protocol, and confidentiality must be maintained. Data/samples must be protected by reasonable security, safeguarding against risks as their loss or unauthorized access, destruction, used by others, and modification or disclosure of data. Data/samples should not be disclosed, made available to or use for purposes other than those specified in the protocol, and shall be preserved for a period, as specified under NSU policy/practices.
9. You shall promptly and fully comply with the decision of IRB to suspend or withdraw its approval for the research protocol.
10. You shall report progress of research to the IRB on annual basis.

I wish you success in running the above-mentioned study.

cc:
1. Dean, SHLS
2. Chair, Department of Public Health
3. Recording Secretary, NSU IRB/ERC
